# Methodological considerations in the design of trials for safety assessment of new drugs and chemical entities

**DOI:** 10.1186/1468-6708-6-1

**Published:** 2005-02-03

**Authors:** Cornel Pater

**Affiliations:** 1 Cornel Pater, Hannover, Germany

## Introduction

Assessment of the QT interval started to receive increased regulatory attention in the late 1980s.

The heightened safety concern was precipitated by repeated reports on torsade de pointes (TdP) and other arrhythmias occurring in patients treated with an antihistamine drug (terfenadine) [1]. ECG measurements performed during the clinical development process by cardiologists (allegedly using "eyeball/calliper" techniques), have failed to identify drug-related QT prolongation. The false-negative conclusions consequently provided have resulted in a number of serious adverse events and, ultimately in the removal of terfenadine from the US market [2].

Similarly, in early 1990s, attempts to decrease sudden cardiac death by novel antiarrhythmic drugs (Cardiac Arrhythmia Suppression Trial – CAST), have demonstrated that a certain degree of arrhythmia suppression was paralleled by a proarrhythmic effect, translated in 3-fold increase in mortality rate among patients treated with encainide or flecainide [3].

Awareness about the potential risk of drug-induced QT prolongation and subsequent risk of malignant arrhythmias has increased gradually since then and, particularly during the past years, regulatory requirements for short- and long-term safety of any new chemical entities have become more stringent. For example, after CAST, the FDA changed its advice regarding antiarrhythmic drugs and required evidence showing minimally, that a new antiarrhythmic agent did not cause death in patients.

The first regulatory guidelines regarding clinical evaluation of the QT/QTc interval prolongation in the context of new drug development were issued in 1997 by the Committee for Proprietary Medicinal Products (CPMP) [4]. Most recently, the FDA has issued its 4^th ^draft document on the matter, clarifying the specific safety issues related to QT/QTc prolongation [5].

## Congenital Long QT Syndrome

The frequency of congenital long QT syndrome (LQTS) is unknown, but appears to be a common cause of sudden and unexplained death in children and young adults. It is much more common than previously thought – possibly as frequent as 1 in 5,000, and may cause 3,000 – 4,000 sudden deaths in children and young adults each year in US [6]. It is present in all races and all ethnic groups, but it is not certain if the frequency is the same in all races.

Clinically, the diagnosis of LQTS is suggested by the occurrence of syncope, cardiac arrest or sudden death [7]. The diagnosis is established on the basis of prolonged QT interval on the ECG. A clearly prolonged QT interval is present in 60% to 70% of affected persons, but the QT is normal or only borderline prolonged in 30–40% of those affected. Overall, about 12% of LQTS patients have a normal QT interval on their baseline, resting ECG.

Torsade de pointes (TdP) tends to appear during exercise (especially swimming) or psychological stress in LQT1, during stress or startle (particularly auditory stimuli) in LQT2 and during rest in LQT3 [8]. Studies on transmural dispersion of repolarization (TDR) in experimental models have shown it to be linked to the genesis of TdP. TDR has different features in the three different forms of LQT referenced as LQT1, LQT2 and LQT3 [9].

### Diurnal and sex-related pattern of QT interval

The maximal QT interval over 24 hours in normal subjects is longer than thought so far (440 ms). Both QT and QTc intervals are longer during sleep. The QT interval and QTc variability reach peak shortly after awakening, which may reflect increased autonomic instability during early waking hours. The time of the peak value corresponds to the period of reported increased vulnerability to ventricular tachycardia and sudden cardiac death. These findings have implications regarding the definition of QT prolongation and its use in predicting arrhythmias and sudden death [10].

At rest, the surface ECG in women displays longer QT interval [11], lower T wave amplitude [12] and less QT dispersion [13]. The QT interval displays greater shortening during exercise as compared to men, as a consequence [14]. Women are also known to have a greater propensity towards developing TdP when treated with agents belonging to class III antiarrhythmic drugs [15,16]. Besides, women are more susceptible to development of malignant arrhythmias in various settings of QT prolongation [12]. The basis for sex differences in repolarization appear to be, at least in part, influenced by sex hormones [17].

However, most recent data derived from a novel, automated QT-analysis algorithm, indicate that there are also sex differences in the dynamics of the QT interval during exercise and recovery in healthy subjects [18]. Women exhibited greater QT-interval shortening during accelerating heart rates and greater QT-interval prolongation during decelerating heart rates than in men. These results suggest that women might have a greater QT interval-rate adaptation, contributing to the greater prevalence of drug-induced TdP episodes in women as compared to men.

In this context, the currently 20 ms sex difference in the rate-adjusted QT interval, recommended by the regulatory agencies, might need to be revised.

### Acquired forms of Long QT interval in diseased patients

It is estimated that more than 50 marketed agents and an equivalent number of drugs under development have been found to block potassium channels, to prolong the QT interval and induce, in some individuals, malignant arrhythmias. TdP is, however, a relatively rare event with a rate of 2–3% for some drugs [19]. Drugs which prolong the QT interval exist in every therapeutic class [20]. An international registry for cases of drug-induced arrhythmias associated with QT prolongation can be found on the web [21].

## The pathophysiology of the TdP

Prolongation of the QT interval on the ECG is caused by increased duration of the action potential (AP) of the ventricular myocytes. Inhibition or activation of the potassium channels in the cells belonging to the different myocardial layers (Purkinje cells, subendocardial myocytes, mid-myocardial M cells and subepicardial myocytes) [22], interferes with the normal repolarization process and triggers different patterns of AP duration. The M cells for example, are characterised by prolonged repolarization in comparison with the epicardial or the endocardial layers.

The potassium channels are of particular importance in drug-related QT changes, most notably the rapid component of the delayed rectifier potassium current (I_Kr_) channel. Blockage of the channel caused by the human ether-a-go-go-related gene (HERG) protein, the gene encoding for the I_Kr_, has been implicated in many of the drug-induced changes.

The model used to explain the increased propensity toward malignant arrhythmias secondary to prolonged QT interval is based on extraneously induced, altered depolarization process with occurrence of "early after-depolarization" action potentials (EADs), which register on the surface ECG as prolonged QT interval [23].

**Figure 1A:**
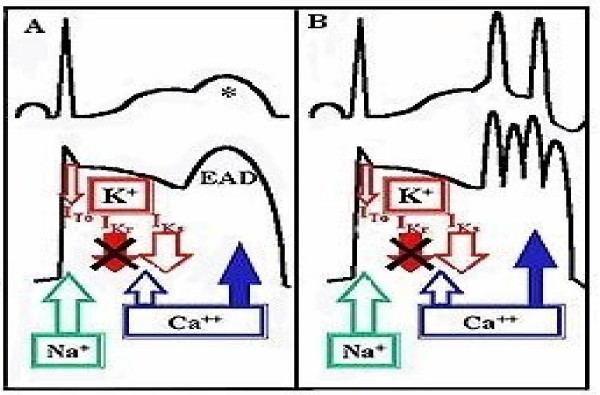
"Humps" on the terminal part of the T-wave reflecting early ADPs. **Figure 1b** EAD degenerating in tachycardia.

In the drug-induced model, any drug, normally used for therapeutic purposes, but which interferes with the inward/outward ion currents across the cell membrane is leading to a prolongation of the action potential duration (APD) and thereby delayed repolarization. Certain drugs have the property to block the potassium channels (I_Kr_) in order to achieve a desired antiarrhythmic effect. These types of changes facilitate additional inward Ca^++ ^currents that further prolong the action potential. Consequently, the AP not only fails to repolarize but also depolarizes again, creating characteristics "humps" which, actually are EADs (Fig. 1A) [24].

Genetic defects of the Na^+ ^or of the K^+ ^channels lead to lengthening of QT interval and EADs which may trigger ventricular extrasystoles (VES). Occurrence of burst-like, repetitive EADs may degenerate in a tachycardia (see Fig. 1B) with particular features, termed torsade de pointes [25]. The French term torsade de pointes, suggests a rapid polymorphic tachycardia in which the QRS axis rotates 360 degrees over a sequence of 5 to 20 complexes [15].

Such early EADs also occur in a multitude of cases such as: bradycardia, diuretic-induced hypokalemia or hypomagnesemia, treatment with natrium or calcium channel blockers.

Preferential prolongation of the action potential duration in the M cells is thought to underlie QT prolongation, the phenotypic appearance of abnormal T-waves, the pathologic U-wave, and the development of TdP.

It is generally accepted that a focal activity initiates the onset of TdP, whereas functional re-entry is responsible for its maintenance [26].

Results from more recent research (27) suggest that changes in a new variable termed "T-wave peak to T-wave end" interval (TPE) would predict increased risk in subjects with LQT1 and LQT2. These changes would reflect the dynamicity of the transmural dispersion of repolarization (TDR) in clinical setting, in LQTS patients. Increased TPE interval may show to be the electrophysiological substrate for TdP. Modulation of the TPE interval magnitude seems to be a property of the *Iks *and *Ikr *(rapidly respectively slowly activating delayed rectifier potassium current) defects. The TPE interval, as an index of TDR, has been proved to be clinically useful in assessing arrhythmic risk [28-31].

## Current regulatory recommendations. Objectives and Scope

Drugs with significant effects on repolarization must be identified and their risk quantified in preclinical and clinical development. Risk-benefit assessment of drugs under development, with particular emphasis to their propensity to prolong repolarization should be individualized to their pharmacokinetic and pharmacodynamic profile as well as to their safety characteristics. The following major aspects need to be addressed:

• Rigorous assessment of the agent's effects on the QT/QTc interval.

• Assessment of the QT/QTc prolongation-related safety risks of the particular drug against its potential benefits.

The above-mentioned issues should be taken into account in any of the following circumstances:

• Development of a novel agent (with non-antiarrhythmic properties).

• A marketed agent for which new dose or route is being developed (with consequent potential increases in pharmacokinetic parameters – Cmax, AUC values).

• A marketed agent for which a new indication or new target patient population is pursued.

• A marketed agent belonging to a chemical or pharmacological class in which any other drug may have been associated with any of the following events: QT/QTc prolongation, TdP or sudden death during postmarketing surveillance.

### The "thorough QT/QTc study" – General Considerations

The "thorough QT/QTc study" is about to emerge as the comprehensive "clinical data set" fully complying with current regulatory requirements, as opposed to "non-clinical" testing that may, or may not generate sufficient information considered to preclude risk of QT/QTc prolongation. Judgement is required on a case-to-case basis on whether the "clinical data set" following completed non-clinical testing [32,33] is still necessary or not, with correspondent adjustment of study design variables.

Being considered a biomarker of proarrhythmic risk, the QT/QTc interval is the pharmacodynamic (PD) parameter which is explored to assess drug induced changes in heart rate (HR) and ECG parameters as correlated to plasma drug concentrations (PK). The PK/PD analysis implies a standardized collection of blood samples for determination of PK parameters (Cmax, Tmax, AUC) and recording of 12-lead ECGs for measurement and computation of specific ECG parameters (PR, QRS, QT/QTc). All of these measurements can be expected to show exposure-response relationships that enhance comparison of the investigated drug with its comparator (placebo or active), primarily with respect to its safety.

**Figure 2A:**
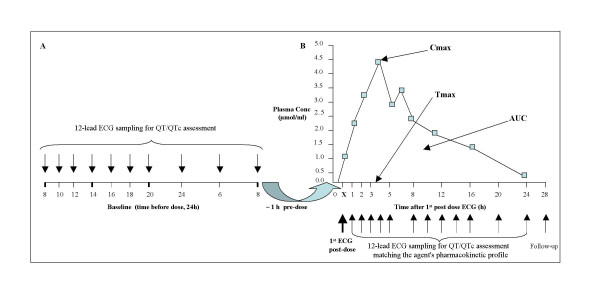
12-lead ECG sampling pattern in the run-in period (baseline). Minimum number of recordings are highlighted at 8, 14 and 20 hours. **Figure 2B** Plasma concentration-time profiles (PK/PD analysis).

In early phase I studies, when the PK profile of the drug is eventually still unknown, a traditional PK study should be performed with the aim to determine the *plasma concentration-time profiles *(see Fig. 2B). This allows not only calculation of AUC but also determination of concentration versus time profiles over a dosing interval for each individual, as well as for the population. This approach yields relatively detailed exposure information that can be correlated to the observed responses in individuals. The exposure-response relationship based on concentration-time profiles can provide time-dependent information that cannot be derived from AUC or Cmin.

Within the bounds of maintained safety and tolerability, the QT/QTc evaluation should also be performed on ECGs recorded at substantial multiples of the maximum therapeutic exposure (multiples of Tmax – see Fig. 2B – samplings at 8, 12, 16, 20 and 24 hours from Tmax).

**Figure 3A:**
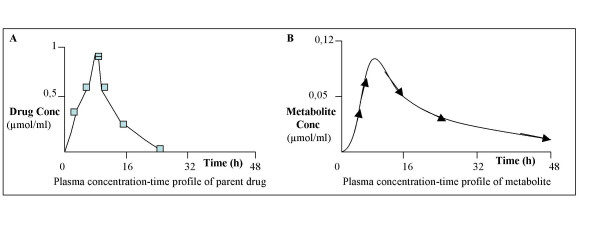
Plasma concentration-time profile of parent drug. **Figure 3B** Plasma concentration-time profile of metabolite.

Knowledge of "concentration increases" that might occur due to drug-drug or drug-food interactions require specifically adjusted study designs. Likewise, in instances where increased plasma concentrations and ensuing QT/QTc prolongation effects may occur due to a metabolite with different pharmacokinetic profile from that of the parent drug (see Fig. 3A and 3B), a tailored study design will be needed.

When the "through QT/QTc study" assessment is intended to be performed in drugs with well known pharmacokinetic profile (known optimal dose and therapeutic window), the sampling plan for both blood collection and ECG recordings will be spaced to match the known peak plasma concentration (Cmax – for single doses of the formulations) or the attained "steady state" after multiple dosing. Importantly, there can be large interindividual variability in the time to peak concentration with differences in the PK profile (e.g., Tmax, time to Cmax) due to demographics, disease states, etc., compelling to closely spaced samplings to account for these differences.

## Study design – Methodological Considerations

The study design needs to be adjusted to the individual drug's pharmacokinetics and safety characteristics. Both the crossover and the parallel designs have specific advantages and relative disadvantages that need to be taken into account in the particular case at hand (see Additional file 1). However, as a rule, regardless of the design alternative chosen, the study should be *randomised*, *double-blind and controlled*.

### Primary objectives

To quantify the dose-, concentration-, and time-relationships of the drug on the QT/QTc interval in the target population at therapeutic and supra-therapeutic plasma concentrations.

### Secondary objectives

Collection of (serious) adverse events such as:

• Absolute QT/QTc prolongation: - QTc > 500 ms and/or

- QTc > 60 ms increase as compared to baseline

• Events suggestive of arrhythmia:- TdP

- Cardiac arrest/Sudden death

- VT/VF

- Syncope

- Dizziness

- Seizures

- Palpitations

### Selection of control

Selecting a control for the purpose of demonstrating safety of a product in terms of "no risk for QT/QTc prolongation effect" is inevitably facing the question of whether an active control or a placebo should be used. The basic assumption is however, that the largest time-matched mean (baseline subtracted) difference between the drug and control (placebo or active control) for the QT interval is ≤ 5 ms, with a one-sided 95% CI that excludes an effect at <8 ms.

Placebo-controlled trials are still used to demonstrate effectiveness of new drugs and, for circumstance in which no increased risk for patients is foreseen, use of placebo seems appropriate and ethical, provided that the patients are fully informed and that they give written, informed consent [34-36].

At closer scrutiny, use of placebo in the particular case of demonstrating safety of a drug is not, by far, so much ethically charged as usually, as the subjects supposed to receive it are, in fact, exposed to a lesser degree of risk by QT prolongation. On the other hand, the "placebo-induced changes" are a reflection of underlying variability of the QT/QTc, an otherwise a well-known phenomenon. With other words, use of placebo-control in these cases is fully justifiable.

Use of an active control, while extensively advocated currently, due to its considerable credibility as compared with placebo, may raise troublesome methodological issues, typical for equivalence trials, such as the rigorous choice of control agent with special emphasis to essay sensitivity, etc.[34]. Commonly, the primary objective of an equivalence/noninferiority trial is to demonstrate that the efficacy of a new treatment matches that of the control treatment while, in the "thorough QT/QTc study", the goal is to demonstrate that the safety of the new drug is equal or at least not worse than that of the control agent. This translates into the need to demonstrate that the new agent doest not prolong the QT/QTc by more than 5 ms on average, as compared to the control. That is to say that the "equivalence margin" is set to 5 ms, although, a definite prolongation effect will be stated if the upper bound of the one-sided 95% CI would exceed 8 ms [5].

The 5 ms value, as an average threshold for demonstrating non-inferiority of the tested drug versus a comparator, might be a too ambitious cut-off point (i.e., too low). With the technique currently available, this level of accuracy may possibly be attained by some highly skilled analysts, but it might be difficult to be maintained as an average level for an entire group of analysts. A more reasonable and practically attainable average value would be 10 ms. Individual subjects displaying prolongation in excess of 10 ms would, however, need to be given careful scrutiny.

### Target patient population

These studies are generally performed in normal, healthy, adult volunteers. The subject population should be selected carefully to minimize inter-subjects variations.

Restrictive eligibility criteria are recommended in early phase studies (I and II) of compounds known to have APD or QT prolonging effects, with subsequent widening of criteria in later phase studies (II and III).

It is estimated that ECGs should be generated in at least 100 volunteers (including females and males), for NCE with no pre-clinical evidence of QT prolongation [37], and in at least 200 volunteers (including females and males) for NCE with pre-clinical evidence of prolonged action potential duration or prolonged QT/QTc [37].

The test and the reference products are usually administered to the subjects in the fasting state (overnight fast for at least 10 hours). These subjects should not take any other medication for one week prior to the study or during the study. Identical test conditions must be used for the two group subjects with respect to: foods, fluid intake, physical activity, posture, etc. and, the physical characteristics of the subjects should be standardized (age, height, weight, and health) [38].

Clinical studies in later phases of development (phase III) and after market approval (phase IV) are supposed to have enlarged inclusion criteria to encompass female and elderly patients, patients with associated comorbidities and with concomitant treatment. Exposure to the relative new treatment of a heterogeneous population, to mimic the real population anticipated to be the end-user of the drug in the future, is meant to create a "worst case scenario" for drugs that in the pre-clinical and clinical development stages have shown effects on the QT/QTc interval. Establishing with confidence the behaviour of the QT/QTc interval in these patients, while exposed to the peak effect (Cmax/steady state) of the drug, is not only an effective risk management tool but also a highly ethical issue.

## Timing of ECG recordings

### Baseline ECG sampling

For NCE with suspected, or known from previous clinical studies, effects on the HR and/or APD, 10 to 20 baseline ECGs are required (see Fig. 2A). For agents administered intermittently, repeated baseline ECG assessments may be needed prior to each new treatment period. Carry-over effects should be carefully taken into consideration when cross-over design is employed.

During the run-in period of later phase trials (II-III), at least three baseline ECGs should be recorded [39].

### "On-treatment" ECG sampling

The pattern of ECG sampling should match the planned blood sample collections for PK assessment (see Fig. 2B). There will be a few or up to 20 ECGs recorded during 24 hours period, depending on how the PK/PD analysis has been planned to be performed, on the amount of knowledge regarding the agent's pharmacokinetics as well as on the information generated by previous pre-clinical studies.

However, regardless of the study design, whenever possible, ECGs should be recorded at the same time of the day during both baseline period and after randomisation (during "on-treatment") to minimize the confounding effects of diurnal variations and postprandial effects [37].

For drugs with known metabolite(s), the ECG recordings should cover the prolonged blood sampling for the plasma concentration-time profile of the metabolite (see Fig. 3B).

Whenever ethically justifiable, for the case of inadvertent over-dosage or metabolic inhibition, it is recommendable that ECGs should be recorded at substantial multiples of the maximum therapeutic exposure, even in excess of the upper bound of the anticipated therapeutic range (see Fig. 2B – sampling at 28 hours).

### Measurement of QT interval

Quality of ECG recordings is of paramount importance for the reliability of the data generated. Poor quality traces due to artefacts or lead misplacement should be avoided through appropriate training of the staff in charge with acquisition of ECGs. Whether these people are professionals or temporary research staff, all are supposed to have a high level of expertise in ECG acquisition technique and be able to validate tracings that are analysable or not.

Standard 12-lead ECGs should be taken in supine, after at least 5 minutes rest with default calibration of the recording device at 1 mV, speed at 50 mm/s.

Currently, standard lead II is chosen for measurement of RR and PR interval, QRS complex and the QT interval, on at least three cardiac cycles. Two additional precordial leads may be used for performance of the same measurements (e.g., V3-V4). Means are computed consequently, from one or three leads.

Manual measurement of different ECG parameters is charged with problems of accuracy and reproducibility due to the inter- and intra-observer variability inherent in such highly demanding tasks while, interpretation of ECG tracings is known to vary from one clinician to another [40].

However, ICH-GCP-compliant quality control and quality assurance SOPs, as well as systematic performance analyses applied to the individual analysts/technicians and their output data, employed nowadays in certain core laboratories, ensure the prospective clients of minimized inter- and intra-observer variability regarding the measurements performed and of high level of accuracy of the output results in the range of ± 10 ms, around a selected/agreed "gold standard" [41].

In order to ensure an overall high level of performance within a group of technicians/analysts who perform the factual measurements on ECG tracings, performance analysis applied to the group and each individual member of the group, should be run at six months interval. Deviation in the measurements performed of more than ± 10 ms should be addressed speedily and corrective measures implemented. Such quality-assurance performance analyses may maintain a high level of measurements' homogeneity and ensure a high quality of the data provided.

Likewise, ECG tracings as well as summary data are subject to interpretation and reporting by qualified cardiologist(s) [4].

**Figure 4:**
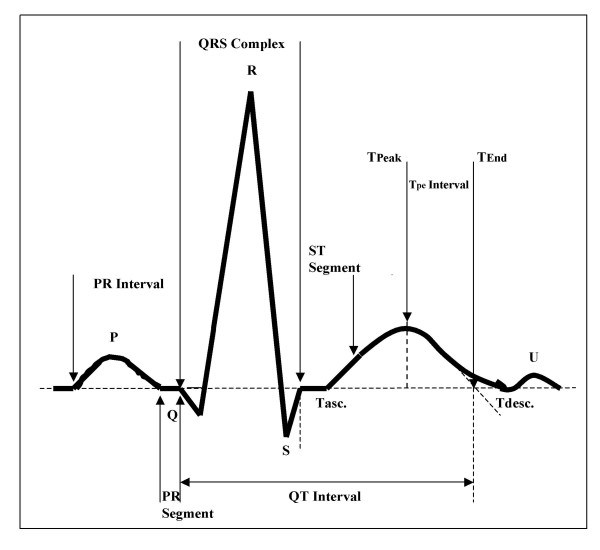
Normal ECG highlighting the common parameters measured when assessing the QT/QTc interval.

Fig. 4 depicts a normal ECG with the most common parameters measured in the process of exploring any new NCE's effects on the QT/QTc interval. Apparently, measuring the QT interval should be a quite straightforward task, however, in practice there are a number of pitfalls and difficulties [30,31].

The beginning of the QRS complex is best determined in a lead with an initial "q" wave – commonly standard lead I or II, and leads aVL, V5 and V6. Sometimes, the "q" wave may be missing (the initial part of the QRS complex is isoelectric) due to its incorporation within the PR interval.

Determining the precise end of the T wave may be simple, when a tangent line to the steepest part of the descending portion of the T wave is drawn and the intercept between the tangent and the isoelectric line is indicating the end of T wave. At times, however, the T wave may be obscured by a superimposed U wave or, in the case of sinus tachycardia, by the ensuing P wave, making the positioning of the fiducial point difficult.

The U wave deflection is usually minimal or isoelectic in lead aVL. The aVL lead is therefore a useful for QT measurement since the end of the T wave is least likely to be obscured by a U wave.

### TU morphology assessment

Different repolarization properties among the epicardium, M cells, and endocardium, as well as their interplay, are responsible for various morphologies of the T-wave and the pathologic U-waves. The T-wave is a symbol of the transmural dispersion of repolarization.

Several hypotheses have been proposed to explain the genesis of the U-wave, which represents the last repolarization component of the ventricules [42] however, the hypothesis that the Purkinje network is responsible for the physiologic U-wave seems most plausible.

Morphology changes of the T and U-wave should be interpreted as warning signs of TdP. Sometimes, a clear demarcation between the two waves is very difficult, exposing to the risk of underestimating the QT interval and, ultimately, to missing the clinical significance of the changes *per se*. Clearly, both qualitative and quantitative assessments of the repolarization changes occurring with different degrees of merger between the T and the U-wave are subject to a certain degree of subjectivity of the assessor. Therefore, it is recommendable that TU-wave morphology assessment to be made by qualified cardiologist(s) according to a standardised methodology. Additional file 2 captures the possible changes that may be encountered in the T-waves, U-waves and different forms of TU mergers in a particular individual. Additional file 3 summarizes the frequency distribution of TU morphology changes across two groups compared.

**Figure 5:**
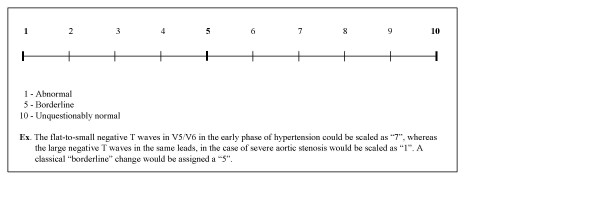
Visual Anlogues Scale to assist in reconciliating the inter-observer assessment of TU morphology.

Given the high level of subjectivity inherent in this type of assessments, with considerable discrepancies between two assessors, even when identical data are assessed, an overall, reasonable conclusion on the TU morphology changes can be provided by use of a visual analogue scale (see Fig. 5). The degree of normality/abnormality in a particular case is estimated on a scale from 1 to 10, on which: "1" – is definite abnormal and "10" – is unquestionably normal. As an example, the flat-to-small negative T-waves in V5/V6 in the early phase of hypertension could be scaled as "7", whereas the large negative T-waves in the same leads, in the case of severe aortic stenosis, would be scaled as "1". A classical "borderline" change would be given a "5".

### QT dispersion (QTD)

Increased dispersion on the QT interval of the electrocardiogram has been proposed as a marker for increased risk of arrhythmias in patients with hypertrophic cardiomyopathy [48], long QT intervals [44], and sustained ventricular arrhythmias [45]. Most of the studies exploring QT dispersion were small and, thereby could not provide accurate data for the sensitivity and specificity of the method to be derived. One study has assessed different cut-off values for QT dispersion by employing ROC analysis, however, the QT dispersion analysed was essentially developed on the basis of a training set [46]. The average normal value of QT dispersion in normal subjects was ≤ 40 ms in 13 studies and ≥ 40 ms in eight studies [47]. The Rotterdam study reported QTc dispersion > 60 ms in apparently healthy subjects aged ≥ 55 years in whom a two-fold increase in sudden death was registered subsequently [48].

Despite sophisticated methods of computerised measurements of QT dispersion [49,50], the reliability of both manual and automatic measurement of QT dispersion is low and the method is considered a crude measure of the abnormalities during the whole course of repolarization [51]. However, more recent studies [52,53] indicated that dispersion in repolarization may arise from differences in the action potential durations between cells situated in difference myocardial layers and that heterogeneity in repolarization might be linked to induction of ventricular fibrillation [42].

The analysis of repolarization variability is commonly based on methods that evaluate spatial and temporal QT dispersion. Recent experimental studies [54] in arterially perfused canine left ventricular wedge preparations, suggest that the second part of the T wave represents the arrhythmogenic substrate and that the peak-to-end interval of the T wave is the trasmural dispersion of the repolarization. The TPE interval of the T wave is postulated to reflect the transmural dispersion in humans (as measured in V5) and might become a parameter to be routinely measured in the future. It is claimed that TPE correlates better than the QT-dispersion with TdP and that a TPE > 280 msec may be useful in predicting risk of TdP in acquired LQTS.

### Heart rate correction of QT interval

The length of the QT interval varies inversely with heart rate and therefore shortens as the heart rate increases. Due to the known substantial inter-subject variability of the QT/RR interval relation, there is no mathematical formula to fit every individual. A formula that performs well in one healthy individual may not do so in another, resulting in over- or undercorrection of the QT interval.

Several correction formulas exist. The Bazett formula (square root – QTcB = QT/RR^1/2^) [55,56], most commonly used, is known to overcorrect at high heart rates and undercorrect at low heart rates [57,58]. The Fridericia formula (cubic root – QTcF = QT/RR^1/3^) [59] is considered to reflect a more accurate correction factor in subjects with tachycardia.

A more recent formula is the Framingham linear correction (QTcL = QT + 0.154 × [1 - RR]) known to be derived from a large patient population and thereby to be considered the most rigorous from an epidemiological perspective [60,61].

The main limitation in the aforementioned formulas is that each of them attempts to correct for heart rate only, while leaving into play a number of other known confounders (diurnal variability, effect of physical exercise, etc.). Disappointingly, analysis done on ECGs sampled from periods of stable heart rate provided no better results [62]. According to Malik et al., the relation between QT interval and heart rate is highly individual [63]. Using a parabolic heart rate correction formula (QTc = QT/RR^α^) they demonstrated a large variability of the α exponent (range: 0.233 – 0.485) in 50 healthy subjects. The same parameter in Fridericia's and Bazett's formulas is 0.33 and 0.50 respectively. Malik and colleagues concluded that correction of QT interval by heart rate may be misleading, regardless of the method used.

QT/RR regression models [64,65] can be used for computing the "right formula for the right data" in experimental situation, however, for practical purposes the Bazett and Fridericia as well as the linear corrections are preferred at present (from regulatory point of view).

### Reporting of measurement results

Reporting of results becomes mostly informative if tabular frequency distribution and frequency histograms are used to display PR, QRS and QTc data (QTcB, QTcF, QTcL) for individuals and/or groups. For the hypothetic example captured in Fig. 2B, tabular representation of the data might be used to illustrate the frequency distribution of a number of parameters (PR, QRS, QTcB, QTcF, QTcL) matching the PK sampling (see Additional file 4). Summary data for the same parameters (Min, Max, Mean) as compared to baseline can be displayed for individual subjects and/or group of subjects (see Additional file 5). The relevant normal ranges for all parameters are given in Additional file 6.

Additional file 7 captures the *baseline*, *mean *and *mean maximum *values for all parameters measured/computed for one group (PR, QRS, QT, QTcB, QTcF and QTcL) and displays the difference (D1) between the *mean *value of each parameter "on-treatment" and the corresponding *mean *value at baseline. Given that a D2 value is to be computed for the second group (comparator), their difference (D2 – D1), for all parameters and the resulting p value (Bomferoni adjusted) could be displayed in Additional file 8.

## Risk assessment as related to prolonged QT/QTc interval

Risk-benefit assessment with respect to a drug's propensity to prolong the QT/QTc interval entails a careful judgement of the frequency and magnitude of QT changes encountered in the preclinical and/or clinical program and balancing the potential risks against the drug's benefit.

The large variability in the prolonged QT/QTc behaviour as to the potential risk for a TdP ensuing, makes this task difficult and requires individual characterisation of a specific drug's effects on repolarization.

Amiodarone, for example, is known to prolong repolarization but to cause rarely TdP. Sotalol which prolongs repolarization through the same mechanism of action as Amiodarone (blockade of the IKr channel) causes a more frequent occurrence of TdP [66].

Some agents may cause slight QTc prolongation but when combined with other drugs that inhibit the metabolism of the suspected drug (e.g., terfenadine and cisapride), marked prolongation can occur [67]. A typical example is dofetilide, a potent QT-prolonging class III antiarrhythmic agent indicated for atrial fibrillation. Concomitant administration of cimetidine with dofetilide was shown to enhance the QT-prolonging effect resulting in a dose-dependent, baseline-related QTc increase of 22% and 33% with 100 mg and 400 mg of cimetidine respectively [68].

It is estimated that about 40–50% of the cases of drug-induced QT interval prolongation and/or TdP, result from drug-drug interactions with metabolic inhibitors (as in the example of dofetilide-cimetidine) and that only 10% are associated with electrolyte imbalance, some 10% with concurrent use of other QT-prolonging drugs and approximately 10–20% of cases have no obvious risk factors [69].

As a general rule, it is recommended that any prolongation should be considered as a potential toxicity [36]. In this context, it has become a widespread consensus that outliers with QTc > 500 ms or a baseline-related increase of QTc > 60 ms are better predictors than the mean QTc values [44]. In such instances, a careful screening for associated underlying risk factors or concomitant drugs is recommended, in order to determine the best course of action. Small QT prolongations (<10 ms) are acceptable as long as there are no associated risk factors. Longer QTc, however, requires individual monitoring and withdrawal from study should be considered, while further elective investigation should be scheduled on a case-to-case basis (see Additional file 9).

### Risk management for marketed products

Ideally, therapy should be individualized on the basis of patient's genotype/phenotype determined through pharmacogenetic studies performed in the early stages of a drug's development and through application of that information while exploring the drug's pharmacokinetic and pharmacodynamic properties, its drug interaction potential as well as when ethnical-based bridging data is generated.

While genotyping of individual cases, where prior informed consent is obtained, based on strong suspicion of genetic substrate having caused substantial QT/QTc prolongation is highly recommendable (such as, outliers in phase I-III studies, patients withdrawn from study due to lack of efficacy or due to type A adverse events), large-scale genotyping in early stages of drug development or pre-prescription genotyping are still controversial.

Consequently, the clinical and scientific community is facing the need to apply classical "individualizing therapy" approaches [70] in reducing the clinical risk of QT/QTc-related adverse events (TdP, VT/VF, sudden death, etc.).

Obviously, the most elementary requirement in this respect is that prescribing physicians should fully comply with contraindications regarding co-prescription of interacting drugs and with the recommendation on appropriate monitoring of targeted patients. More specifically, attention should be given to pharmacokinetic and pharmacodynamic factors that constitute important risk factors [4].

Liver and/or renal diseases act as risk factors at pharmacokinetic level. Likewise, a multitude of metabolic inhibitors (see Additional file 10), when temporarily co-administered, develop high plasma concentration of the parent drugs, exposing them to high-dose pharmacology of the drugs concerned [4].

Pharmacodynamic risk factors include diseases that are associated with QT interval prolongation (see Additional file 11).

Obviously, appropriate monitoring is a *sine qua non *condition for preventing SAE in patients known to be treated with QT-prolonging drugs. QT interval should be monitored in these patients: (i) at baseline; (ii) at steady-state post-dose and at each incremental dose; (iii) when there is an inter-current change in level of risk, and (iv) if the patient develops symptoms of tachycardia or impaired cerebral circulation [4]. Treatment should be discontinued if QTc ≥ 500 ms and appropriate measures instituted based on the clinical picture at hand.

Occurrence of typical AE suggestive of eventual QT-prolongation, should prompt careful investigation of this possibility even in cases where initial QT/QTc assessment has shown to be negative. In such instances, it is recommended that screening for risk factors shall be employed and genotyping performed after receipt of informed consent. Furthermore, consideration should be give to "re-challenge" with the investigational drug under appropriate monitoring conditions, with the aim of obtaining an accurate assessment of the situation at hand as well as for getting useful information on dose- and concentration-response relationship.

## Conclusions

Compelling evidence has accrued during the past years on the potential of several cardiac and non-cardiac drugs to prolong cardiac repolarization (reflected as prolonged QT on surface ECG) and to predispose to life-threatening arrhythmias.

This evidence has a major impact on the risk-benefit ratio of any drug, currently carefully considered from early stages of clinical drug development by pharmaceutical companies, by ethics committees as well as by regulatory agencies.

The broad spectrum of risk factors that may interplay in the increased propensity toward malignant arrhythmias of any new chemical entity is just increasing (congenital LQTS, genetic substrate, comorbidities, concomitant treatment) and adding to the complexity of the problem.

This calls for standardized methodologies to deal with the multifaceted aspects that the QT/QTc prolongation poses in practice, meant to ensure that drugs awarded market approval have undergone appropriate quality assurance scrutiny and, where necessary, further post-marketing surveillance is systematically planned and reported on, in a timely manner.

## Competing interests

The author(s) declare that they have no competing interests.

## Supplementary Material

Additional File 1 Frequency distribution of TU morphology changes across two groups. Click here for file

Additional File 2 TU morphology changes in individual subjects. Click here for file

Additional File 3 Frequency distribution of TU morphology changes across two groups. Click here for file

Additional File 4 Frequency distribution of the PR/QRS/QTc(B/F/L) data matching PK sampling (for individuals and/or groups). Click here for file

Additional File 5 Summary of PR/QRS/QTc(B/F/L) data (for individuals and/or groups). Click here for file

Additional File 6 Normal ranges for the PR/QRS/QTc(B/F/L) data and for the QTc(B/F/L) relative changes to baseline. Click here for file

Additional File 7 Frequency distribution of the baseline and on-treatment values pertaining the PR, QRS, QT, QTcB, QTcF and QTcL parameters as well as the D1 difference. Click here for file

Additional File 8 Summary of outcome differences between the two groups regarding key ECG parameters. Click here for file

Additional File 9 Alert criteria based on ECG findings (measurements) and rational for subject withdrawal from study. Click here for file

Additional File 10 Characteristics of the cross-over and parallel study designs. Click here for file

Additional File 11 Disease associated with prolonged QT/QTc interval. Click here for file

Additional File 12 Abbreviations (Not mentioned in the text!) Click here for file
