# Supplementary material for: Methodological considerations in the design of trials for safety assessment of new drugs and chemical entities
Source: Curr Control Trials Cardiovasc Med. 2005 Feb 3;6(1):1. doi: 10.1186/1468-6708-6-1 (PMC549209; doi:10.1186/1468-6708-6-1)
Supplement: Additional File 1 — Frequency distribution of TU morphology changes across two groups. [file 1468-6708-6-1-S1.doc]

| **Study design** | **Characteristics of study design and Exposure-Response Assessment** |
| --- | --- |
| Crossover, fixed  dose, dose  response | - For immediate, acute, reversible responses - Provide both population mean and individual exposure-response information - Safety information obscured by time effects, tolerance, etc. - Treatment by period interactions and carryover effects are possible - Changes in baseline-comparability between periods can be a problem |
| Parallel, fixed  dose, dose  response | - For long-term, chronic responses, or responses that are not quickly reversible - Provides only population mean, no individual dose response - Should have relatively large numbers of subjects (1 dose per patient) - Gives good information on safety |
