# Supplementary material for: Methodological considerations in the design of trials for safety assessment of new drugs and chemical entities
Source: Curr Control Trials Cardiovasc Med. 2005 Feb 3;6(1):1. doi: 10.1186/1468-6708-6-1 (PMC549209; doi:10.1186/1468-6708-6-1)
Supplement: Additional File 2 — TU morphology changes in individual subjects. [file 1468-6708-6-1-S2.doc]

| Subject Ref. | |  | | |
| --- | --- | --- | --- | --- |
| **ECG param.** | **Variable** | | **Baseline** | **On treatment** |
| **T wave** | NORMAL | |  |  |
|  | Polarity (negative) | |  |  |
|  | Biphasic (+ / - or - / +) | |  |  |
|  | Humped | |  |  |
|  | Symmetric/peaked and/or  High amplitude | |  |  |
|  | Low amplitude or flat | |  |  |
|  | Filled | |  |  |
| **U wave** | NORMAL | |  |  |
|  | Polarity (negative) | |  |  |
|  | Biphasic (+ / - or - / +) | |  |  |
|  | High amplitude | |  |  |
| **T/U wave**  **Merger** | NORMAL | |  |  |
|  | Separate | |  |  |
|  | Partial | |  |  |
|  | Complete | |  |  |
|  | | |  |  |
