# Supplementary material for: Methodological considerations in the design of trials for safety assessment of new drugs and chemical entities
Source: Curr Control Trials Cardiovasc Med. 2005 Feb 3;6(1):1. doi: 10.1186/1468-6708-6-1 (PMC549209; doi:10.1186/1468-6708-6-1)
Supplement: Additional File 3 — Frequency distribution of TU morphology changes across two groups. [file 1468-6708-6-1-S3.doc]

| **TU morphology pattern^*^** | **New Drug, n (%)** | **Comparator, n (%)** |
| --- | --- | --- |
| N |  |  |
| B |  |  |
| N + B |  |  |
| T |  |  |
| E |  |  |

^*^ N – normal (no TU morphology changes at all); B – TU changes exclusively at

baseline; B + T – TU changes at baseline and on treatment; T – TU changes only on

treatment; E – TU changes noticed only on follow-up.
