# Supplementary material for: Methodological considerations in the design of trials for safety assessment of new drugs and chemical entities
Source: Curr Control Trials Cardiovasc Med. 2005 Feb 3;6(1):1. doi: 10.1186/1468-6708-6-1 (PMC549209; doi:10.1186/1468-6708-6-1)
Supplement: Additional File 4 — Frequency distribution of the PR/QRS/QTc(B/F/L) data matching PK sampling (for individuals and/or groups). [file 1468-6708-6-1-S4.doc]

| Subject/Gr. Ref. nr. | |  | | |
| --- | --- | --- | --- | --- |
| **Timing of ECG recordings** | | **PR/QRS/QTc (B,F,L) ranges, (s)** | | |
|  |  | **Shortened/Normal** | **Normal/Borderline** | **Prolonged** |
| Baseline (mean) | |  |  |  |
| On  Treatment  ( (post-dose) | X – 1^st^ ECG |  |  |  |
|  | 1 h after X |  |  |  |
|  | 2 h |  |  |  |
|  | 3 h |  |  |  |
|  | 4 h |  |  |  |
|  | 5 h |  |  |  |
|  | 6h |  |  |  |
|  | 8h |  |  |  |
|  | 10h |  |  |  |
|  | 12h |  |  |  |
|  | 14h |  |  |  |
|  | 16h |  |  |  |
|  | 20h |  |  |  |
|  | 24h |  |  |  |
| Post-treat. | 28h |  |  |  |
