# Supplementary material for: Methodological considerations in the design of trials for safety assessment of new drugs and chemical entities
Source: Curr Control Trials Cardiovasc Med. 2005 Feb 3;6(1):1. doi: 10.1186/1468-6708-6-1 (PMC549209; doi:10.1186/1468-6708-6-1)
Supplement: Additional File 5 — Summary of PR/QRS/QTc(B/F/L) data (for individuals and/or groups). [file 1468-6708-6-1-S5.doc]

| Subject/Gr. Ref. nr. |  | | |
| --- | --- | --- | --- |
| **Baseline (mean)** | **PR/QRS/QTc (B,F,L) summary – on treatment values, (s)** | | |
|  | **Min** | **Max** | **Mean** |
|  |  |  |  |
