# Supplementary material for: Methodological considerations in the design of trials for safety assessment of new drugs and chemical entities
Source: Curr Control Trials Cardiovasc Med. 2005 Feb 3;6(1):1. doi: 10.1186/1468-6708-6-1 (PMC549209; doi:10.1186/1468-6708-6-1)
Supplement: Additional File 6 — Normal ranges for the PR/QRS/QTc(B/F/L) data and for the QTc(B/F/L) relative changes to baseline. [file 1468-6708-6-1-S6.doc]

| **PR** | Shortened | Normal | Prolonged |
| --- | --- | --- | --- |
|  | < 0.12 | 0.12 – 0.22 | > 0.22 |
| **QRS** | Normal | Borderline | Prolonged |
|  | < 0.10 | 0.10 – 0.12 | > 0.12 |
| **QTc (B/F/L)** | Normal | Borderline | Prolonged |
|  | < 450 | 450 - 500 | > 500 |
| **QTc (B/F/L) changes relative to baseline** | Normal | Borderline | Prolonged |
|  | < 30 | 30 – 60 | > 60 |
