# Supplementary material for: Methodological considerations in the design of trials for safety assessment of new drugs and chemical entities
Source: Curr Control Trials Cardiovasc Med. 2005 Feb 3;6(1):1. doi: 10.1186/1468-6708-6-1 (PMC549209; doi:10.1186/1468-6708-6-1)
Supplement: Additional File 7 — Frequency distribution of the baseline and on-treatment values pertaining the PR, QRS, QT, QTcB, QTcF and QTcL parameters as well as the D1 difference. [file 1468-6708-6-1-S7.doc]

| Group Ref. nr. |  | | | | |
| --- | --- | --- | --- | --- | --- |
| **ECG parameters** | **Baseline (mean)** | **On treatment** | | **Diff. (D1)^*^** | **p value** |
|  |  | **Mean** | **Mean Max.** |  |  |
| PR |  |  |  |  |  |
| QRS |  |  |  |  |  |
| QT |  |  |  |  |  |
| QTcB |  |  |  |  |  |
| QTcF |  |  |  |  |  |
| QTcL |  |  |  |  |  |

* Diff. (D1) – between mean “on treatment” and mean at baseline (for the New Drug – “D1” or for the

comparator – “D2”)
