# Supplementary material for: Methodological considerations in the design of trials for safety assessment of new drugs and chemical entities
Source: Curr Control Trials Cardiovasc Med. 2005 Feb 3;6(1):1. doi: 10.1186/1468-6708-6-1 (PMC549209; doi:10.1186/1468-6708-6-1)
Supplement: Additional File 8 — Summary of outcome differences between the two groups regarding key ECG parameters. [file 1468-6708-6-1-S8.doc]

| **ECG parameters** | **D1^*^** | **D2^**^** | **D2 – D1** | **p value** |
| --- | --- | --- | --- | --- |
| PR |  |  |  |  |
| QRS |  |  |  |  |
| QT |  |  |  |  |
| QTcB |  |  |  |  |
| QTcF |  |  |  |  |
| QTcL |  |  |  |  |

* Diff. (D1) - between mean “on treatment” and mean at baseline (for the New Drug)

** Diff. (D2) – between the mean “on treatment” and mean at baseline (for the comparator)
