# Supplementary material for: Methodological considerations in the design of trials for safety assessment of new drugs and chemical entities
Source: Curr Control Trials Cardiovasc Med. 2005 Feb 3;6(1):1. doi: 10.1186/1468-6708-6-1 (PMC549209; doi:10.1186/1468-6708-6-1)
Supplement: Additional File 9 — Alert criteria based on ECG findings (measurements) and rational for subject withdrawal from study. [file 1468-6708-6-1-S9.doc]

| **ECG finding/Alert** | **Rationale for withdrawal** |
| --- | --- |
| Absolute QTc prolongation (>500ms)  Relative QTc Prolongation (>60 ms  compared to baseline)  AMI  Conduction disturbances (blocks of  Mobitz type II: 2^nd^ and 3^rd^ degree)  TdP/VT/VF | Risk of TdP  Risk for TdP  Life threatening; requires  investigation  Risk of syncope; requires  investigation  Life threatening; requires  investigation |
