# Supplementary material for: Methodological considerations in the design of trials for safety assessment of new drugs and chemical entities
Source: Curr Control Trials Cardiovasc Med. 2005 Feb 3;6(1):1. doi: 10.1186/1468-6708-6-1 (PMC549209; doi:10.1186/1468-6708-6-1)
Supplement: Additional File 10 — Characteristics of the cross-over and parallel study designs. [file 1468-6708-6-1-S10.doc]

| **Cyp enzyme** | **Distribution** | **Chromosamal location** | **Alleles known (10.04.03)** | **Main QT-prolonging drugs metabolised** |
| --- | --- | --- | --- | --- |
| CYP2B6 | Polymorphic | 19q13.2 | 16 | Efavirenz, methadone |
| CYP2C19 | Polymorphic | 10q24.1-q24.3 | 19 | Nelfinavir, citalopram |
| CYP2D6 | Polymorphic | 22q13.1 | 73 | Most antipsychotics, antidepressants and antianginals, encainide, flecainide, lorcainide, ajmaline, indoramin |
| CYP3A4 | Unimodal | 7q21.1 | 25 | Most class III antiarrhythmics, antihistamines, cisapride, pimozide, ziprasidone, levacetylmethadol, methadone, tacrolimus, tamoxifen, clarithromycin, erythromycin, halofantrine, quinidine, bupivacaine |

Shah RR, 2004
