# Supplementary material for: Methodological considerations in the design of trials for safety assessment of new drugs and chemical entities
Source: Curr Control Trials Cardiovasc Med. 2005 Feb 3;6(1):1. doi: 10.1186/1468-6708-6-1 (PMC549209; doi:10.1186/1468-6708-6-1)
Supplement: Additional File 11 — Disease associated with prolonged QT/QTc interval. [file 1468-6708-6-1-S11.doc]

| Congestive heart failure Hepatic dysfunction  Ischemic heart disease Electrolyte imbalance  Myocarditis Hypothermia  Subarchnoid hemorrhage Liquid protein diets  Mitral valve prolapse Vagotomy |
| --- |
