# Supplementary material for: Methodological considerations in the design of trials for safety assessment of new drugs and chemical entities
Source: Curr Control Trials Cardiovasc Med. 2005 Feb 3;6(1):1. doi: 10.1186/1468-6708-6-1 (PMC549209; doi:10.1186/1468-6708-6-1)
Supplement: Additional File 12 — Abbreviations (Not mentioned in the text!) [file 1468-6708-6-1-S12.doc]

**Abbreviations**

AMI - Acute Myocardial Infarction

AP - Action Potential

APD - Action Potential Duration

AUC - Area Under Curve

Cmax - Maximum concentration

CPMP - Committee for Proprietary

Medicinal Products

EAD - Early after-depolarisations

ECG - Electrocardiogram

FDA - Food and Drug Administration

HERG - Human ether-go-a-go related gene

HR - Heart Rate

Ms - Milliseconds

LQTS - Long QT Syndrome

K^+^  - Potassium ion

Na^+^ - Sodium ion

NCE - New chemical entity

IKr - Delayed potassium rectifier current

QT - QT interval on the ECG

QTc - QT corrected

QTcB - QT corrected by Bazzett formula

QTcF - QT corrected by Framingham formula

QTcL - QT corrected by linear formula

PK - Pharmacokinetics

PD - Pharmacodynamics

TdP - Torsade de Pointes

TDR - Transmural Dispersion Repolarisation

TPE - T-wave peak-to-end

Tmax- The time at which Cmax occurs
